# Fiber Optic Particle Plasmon Resonance Biosensor for Label-Free Detection of Nucleic Acids and Its Application to HLA-B27 mRNA Detection in Patients with Ankylosing Spondylitis

**DOI:** 10.3390/s20113137

**Published:** 2020-06-01

**Authors:** Yen-Ta Tseng, Wan-Yun Li, Ya-Wen Yu, Chang-Yue Chiang, Su-Qin Liu, Lai-Kwan Chau, Ning-Sheng Lai, Cheng-Chung Chou

**Affiliations:** 1 Department of Chemistry and Biochemistry and Center for Nano Bio-Detection, National Chung Cheng University, Chiayi 62102, Taiwan; tsengyentaozzy@gmail.com (Y.-T.T.); smilelili72@gmail.com (W.-Y.L.); wen19860312@hotmail.com (Y.-W.Y.); chiangcy@yuntech.edu.tw (C-Y.C.); 2 Graduate School of Engineering Science and Technology, National Yunlin University of Science and Technology, Yunlin 64002, Taiwan; 3 Immunology and Rheumatology, Department of Medicine, Buddhist Dalin Tzu Chi General Hospital, Chiayi 62247, Taiwan; df897226@tzuchi.com.tw; 4 Department of Biomedical Sciences, National Chung Cheng University, Chiayi 62102, Taiwan

**Keywords:** fiber optic particle plasmon resonance, gold nanoparticle, biosensor, ankylosing spondylitis, HLA-B27

## Abstract

We developed a label-free, real-time, and highly sensitive nucleic acid biosensor based on fiber optic particle plasmon resonance (FOPPR). The biosensor employs a single-strand deoxyoligonucleotides (ssDNA) probe, conjugated to immobilized gold nanoparticles on the core surface of an optical fiber. We explore the steric effects on hybridization affinity and limit of detection (LOD), by using different ssDNA probe designs and surface chemistries, including diluent molecules of different lengths in mixed self-assembled monolayers, ssDNA probes of different oligonucleotide lengths, ssDNA probes in different orientations to accommodate target oligonucleotides with a hybridization region located unevenly in the strand. Based on the optimized ssDNA probe design and surface chemistry, we achieved LOD at sub-nM level, which makes detection of target oligonucleotides as low as 1 fmol possible in the 10-μL sensor chip. Additionally, the FOPPR biosensor shows a good correlation in determining HLA-B27 mRNA, in extracted blood samples from patients with ankylosing spondylitis (AS), with the clinically accepted real-time reverse transcription-polymerase chain reaction (RT-PCR) method. The results from this fundamental study should guide the design of ssDNA probe for anti-sense sensing. Further results through application to HLA-B27 mRNA detection illustrate the feasibility in detecting various nucleic acids of chemical and biological relevance.

## 1. Introduction

The hybridization of nucleic acids to their complementary sequences is a fundamental process during cellular and molecular biology, and has been the key for development of DNA and RNA biosensors. Nucleic acid hybridization, between two complementary strands of nucleic acids, is well-known for their high affinity interaction and a promising tool for clinical, environmental, and food analysis by optical biosensors [1,2,3]. The analysis of gene sequences and gene polymorphisms plays a fundamental role in numerous important applications, such as genetic disease diagnostics, molecular medicine, pathogen detection, genetically modified organism (GMO), drug screening, and forensics. As nucleic acid itself does not provide any signal, current methods of identification of specific DNA sequence in biological samples are based on isolation of double stranded (ds) genomic DNA and further polymerase chain reaction (PCR) to amplify the target sequence of DNA with an appropriate label. However, performing PCR protocols requires tedious procedures of hours, using sophisticated equipment in central laboratories, by skilled personnel, and thus, limits its applicability in becoming rapid point-of-care testing (POCT) methods. Therefore, there is great demand in eliminating the need for labeling and amplification to develop rapid, reliable, and user-friendly biosensors.

Recently, optical biosensors, based on nanoplasmonics, have the potential to realize compact and high sensitivity devices without the use of molecular labels [4,5,6]. Particle plasmons are charge density collective oscillation confined to metallic nanoparticles. These nanoparticles characteristically exhibit a strong extinction band called particle plasmon resonance (PPR) band, also known as localized surface plasmon resonance (LSPR) band. The extinction cross-section and peak wavelength of the PPR band are highly dependent on the local refractive index of the nanoparticle’s surrounding medium, and thus, the binding events to those functionalized nanoparticles [7]. A typical configuration for a nanoplasmonic nucleic acid biosensor consists of a single strand DNA (ssDNA) probe, conjugated to noble metal nanoparticles, which are immobilized on a substrate, where the ssDNA probe hybridizes with its complementary target strand.

The glass slide is a popular choice of substrate for biosensors, based on nanoplasmonics of noble metal nanostructures. However, the signal-to-noise ratio for glass slide-based nanoplasmonic biosensors obtained by either the transmission mode with a single pass of light or reflection mode, with a double pass of light, through a submonolayer of noble metal nanoparticles on a slide that is not high, as a result of the low absorbance of a nanoparticle submonolayer. The absorbance of such a nanoparticle submonolayer can be significantly enhanced by multiple total internal reflections in an optical fiber [8,9]. Based on this concept, a partially unclad optical fiber, with 2-cm sensing region modified with gold nanoparticles (AuNPs), has been developed as a fiber optic particle plasmon resonance (FOPPR) sensing platform, which shows desirable features, such as label-free, real-time, and high sensitivity in biomolecular detection [8,10]. The FOPPR biosensor has been applied to quantitative analysis of protein biomarkers in real samples, and the results agree quantitatively with standard method [11,12,13,14,15]. Various forms of the FOPPR biosensor have also been developed for quantitative measurement of proteins, small molecules, and ions [16,17,18,19,20,21,22,23]. However, to the best of our knowledge, it has not been applied in the detection of nucleic acids.

The efficacy of biosensing depends on the interactions between an immobilized bio-recognition probe and an analyte. In this study, the antisense oligonucleotide probes were introduced to become bio-recognition ligands and applied to the FOPPR biosensor. The factors influencing the immobilization of ssDNA probe to the biosensing efficacy of DNA and RNA were explored. Then, we report the applications of the FOPPR biosensor to detection of H5 RNA and HLA-B27 mRNA.

Avian H5N1 influenza virus is a negative single strand RNA (ssRNA) virus and has received enormous attention as a potential pandemic threat and also a threat to human health. The conserved RNA sequence of hemagglutinin 5 (H5) was used as a model RNA to test our FOPPR biosensor for label-free detection of DNA-RNA hybridization.

The human leukocyte antigen class I gene HLA-B27 is the strongest risk factor for ankylosing spondylitis (AS) [24,25,26], a chronic inflammatory arthritic inherited disorder. HLA-B27 is an HLA class 1 molecule that efficiently binds and presents immuno-dominant peptide epitopes to cytotoxic T cells in several important viral infections, including influenza, HIV, EBV and hepatitis C [27]. We had the exon 1 and exon 2 mRNA sequence of HLA-B27 as our target to establish a FOPPR biosensor with an antisense ssDNA probe for detection of mRNA.

## 2. Materials and Methods

### 2.1. Reagents and Materials

All reagents were used as received. Cetyltrimethylammonium bromide (CTAB) and ethylenediamine-tetraacetic acid (EDTA 99%) were purchased from Acros (Geel, Belgium). Hydrogen tetrachloroaurate (III) hydrate (HAuCl_4_·xH_2_O) was purchased from Alfa Aesar (Tewksbury, MA, USA). Toluene and potassium phosphate monobasic (KH_2_PO_4_) were from Honeywell Riedel-de-Haën (Morris Plains, NJ, USA). Hydrogen peroxide (30% in H_2_O), potassium chloride (KCl), calcium chloride (CaCl_2_), magnesium chloride anhydrous (MgCl_2_), and D(+) sucrose for analysis were purchased from Showa (Tokyo, Japan). Sodium borohydride (NaBH_4_), ethanol (>95%), tris(hydroxymethyl)aminomethane (Tris), 2-mercaptoethanol (MCE), 6-mercaptohexanol (MCH) and chloroform (CHCl_3_) were purchased from Sigma-Aldrich (St. Louis, MO, USA). (3-Mercaptopropyl)trimethoxysilane (MPTMS) was purchased from TCI (Tokyo, Japan). All aqueous solutions were prepared with ultrapure water from Milli-Q waterurification system (Millipore, Billerica, MA, USA) with a specific resistance of 18.2 MΩ·cm.

Thiolated single strand deoxyoligonucleotides (HS-ssDNA) as ssDNA probes, unmodified single strand deoxyoligonucleotides as target ssDNAs, and unmodified oxyoligonucleotides as target ssRNA were purchased from MD Bio Inc. (Taipei, Taiwan) (see Table 1), with each tube containing 10 μL of material (100 μM) preserved in −80 °C pH 8.0 TE buffer which was composed of 10 mM Tris and 1 mM EDTA. The oligonucleotides were thawed and diluted with 990 μL buffer before use. Multimode plastic-clad silica optical fiber (model F-MBC) was purchased from Newport (Irvine, CA, USA) with core and cladding diameters of 400, and 430 µm, respectively.

### 2.2. Preparation of AuNP-Modified Optical Fibers

Spherical AuNPs were prepared by chemical reduction method in chloroform [28]. 0.0728 g cetyltrimethylammonium bromide (CTAB) and 1.78 mL of 2.43 mM hydrogen tetrachloroaurate (III) solution were mixed in chloroform with a final volume of 10 mL. The solution was vigorously stirred for 10 min, then 800 μL ethanolic solution of 0.15 M sodium borohydride (NaBH_4_) was added under vigorously stirring for another 30 min. The solution then turned from milky yellow to crimson red. After allowing to stand for 3 h, the solution separated into two layers. The upper layer is a colorless aqueous phase solution and the lower layer is an organic phase chloroform with AuNPs. The solution absorption spectra of AuNPs were measured by a Jasco V-570 UV-Vis-NIR spectrophotometer (Tokyo, Japan) and showed one peak wavelength at 524 nm with absorbance about 1.2 a.u.

A JEOL 1200 EX transmission electron microscope (TEM, Tokyo, Japan) operating at 120 kV was used to obtain the images of the AuNPs. Specimens for TEM were prepared by placing a sample drop on a copper grid and allowing it to dry. Histograms derived from TEM image analysis show that the mean diameter of the AuNPs is 7.7 ± 1.5 nm, as shown in Figure S1a of the Supplementary Material.

The sensor fibers were prepared by removal of cladding (2 cm) in the middle part of an optical fiber with CO_2_ laser engraving (V-460 Universal Laser Systems Inc., Scottsdale, AZ, USA) The partially unclad fibers were immersed in 1% MPTMS/toluene for 12 h to form a mercapto-functionalized self-assembled thin layer. Then the optical fibers were washed sequentially using the following solvents: Toluene, toluene/ethanol, ethanol, and ultrapure water to eliminate the excess MPTMS. The functionalized fibers were then immersed in an AuNP solution another 10 h for preparing AuNP-modified optical fibers.

The images of the AuNPs after sputter coating a thin layer of Pt on the sensor fibers were then taken by a Hitachi 4800I field-emission scanning electron microscope (FE-SEM, Tokyo, Japan) operated at 10 kV. The diameter of at least 100 nanoparticles in each batch was determined from these SEM images to characterize the size distribution of the nanoparticles. A histogram derived from the FE-SEM image analysis shows that the size distribution of AuNPs on the optical fiber is 7.7 ± 1.8 nm, as shown in Figure S1b of the Supplementary Material.

### 2.3. Bioconjufation of ssDNA Probe on AuNP-Modified Optical Fibers

To prepare sensor fibers, the AuNP-modified fibers were functionalized with a HS-ssDNA probe, which was designed to bind specifically with its complementary target ssDNA or ssRNA. An immobilization buffer (IB) containing 1 M KH_2_PO_4_ was used to prepare the solutions of HS-ssDNA probe.

The base sequences of the HS-ssDNA probes for target H5 ssDNAs and target H5 ssRNA are as follows:

Thiolated H5-5′ ssDNA probe (30-mer base with 20-mer adenine spacer)
HS-(CH_2_)_6_-A_20_-5′-TCCCT AGCAC TGGCA ATCAT GGTAG CTGGT-3′
Thiolated H5-3′ ssDNA probe (30-mer base with 20-mer adenine spacer)
5′-TCCCT AGCAC TGGCA ATCAT GGTAG CTGGT-3′-A_20_-(CH_2_)_6_-SH

All stock solutions of oligonucleotides were prepared in TE buffer (pH 8.0) and stored in a −80 °C refrigerator until use. To immobilize a HS-ssDNA probe on the AuNP surface, the AuNP-modified fibers were immersed in a solution of 1 μM HS-ssDNA in the IB for 1 h. Then, the fibers were rinsed with the IB to remove physisorbed HS-ssDNA. Afterward, the fibers were immersed in 1 mM MCE for 5 min and subsequently rinsed with the IB.

### 2.4. Fabrication of Sensor Chips

The microfluidic sensor chip was composed of two cyclic olefin copolymer (COC) plates, a cover and a bottom plate, with dimensions of 2.5 cm (width) × 5.0 cm (length) × 0.2 cm (thickness) and fabricated by an injection molding machine. The bottom plate contained a microchannel with a depth of 800 µm and a width of 800 µm. An inlet and an outlet for sample introduction were connected to two small access holes and mechanically bored into the microchannel, which accommodated a sensor fiber. The total length of the sensor fiber was 8 cm with a sensing zone of 2 cm in the middle. Thus, the free volume of the microchannel was estimated to be 10.3 μL. The cover and the bottom plates were glued by a 3M sticker to form the chip. Teflon tubing was then attached to the chip through both the inlet and outlet. The samples and buffers were loaded into the chips by a syringe pump.

### 2.5. Biosensing System and Measurements

**Figure 1:**
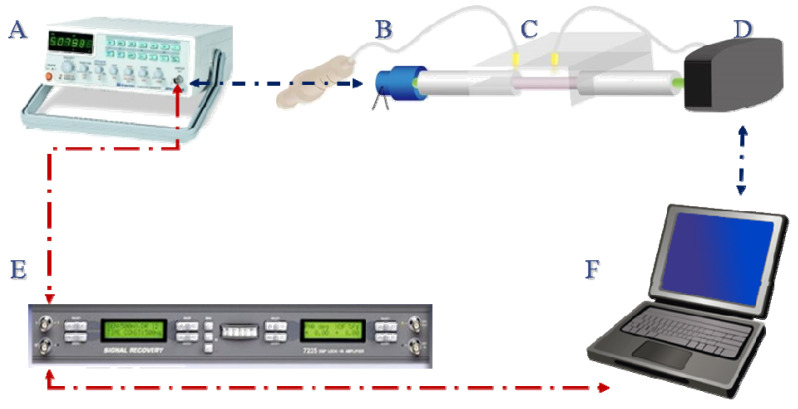
Schematic representation (not to scale) of the experimental setup used for the fiber optic particle plasmon resonance (FOPPR) sensing system. The system consists of: (**A**) function generator; (**B**) LED; (**C**) sensor chip; (**D**) photoreceiver; (**E**) lock-in amplifier; (**F**) computer.

A schematic of our FOPPR biosensing system is shown in Figure 1, which consists of a light-emitting diode (LED, model IF-E93, Industrial Fiber Optic, Inc., Tempe, AZ, USA) with a peak wavelength of 532 nm, a function generator (model GFG-8255A, GW instek, Taipei, Taiwan) to modulate the LED output at a frequency of about 1 kHz, a sensor chip, a photoreceiver (model 2001, New Focus, San Jose, CA, USA), and a lock-in amplifier (model 7220, Signal Recovery, Santa Clara, CA, USA). The strategy of FOPPR sensing is to use a narrow band incident light to excite the plasmon resonance of AuNPs on an optical fiber and then monitoring the intensity of light exiting the fiber. Since the absorption coefficient of AuNPs increases when the AuNP’s surrounding local refractive index (RI) increases [12]. Hence, the increase of local RI at the AuNP surface during molecular binding can be revealed in real-time by a decrease of transmitted light intensity through the optical fiber in form of a molecular binding kinetic curve [29]. In this study, the sensor response is defined as I/I_0_, where I_0_ is the light intensity exiting a sensor fiber which is immersed in a blank and I is the light intensity exiting the same sensor fiber which is immersed in a sample.

A hybridization buffer (HB) at pH 7.4 containing 20 mM Tris, 1 M NaCl, 5 mM KCl, and 1 mM CaCl_2_, and 100 mM MgCl_2_ was used to prepare the samples. The response time of the hybridization between a target oligonucleotides and a ssDNA probe was defined as 95% of the maximum signal changed. To complete the hybridization, the reaction time was allowed to take longer than 15 min after injection of a sample.

All data are reported as the mean ± standard deviation (SD) as described in the text. Regression analysis was used to determine the correlation between the results obtained by the FOPPR biosensor and the RT-PCR method. Statistical analysis was performed using GraphPad Prism and Origin.

### 2.6. Relative Quantitation of mRNA-B27 in Plasma by Real Time RT-PCR

The method of real-time reverse transcription-polymerase chain reaction (RT-PCR) for relative quantitation of mRNA-B27 in plasma was according to a previous work using a pair of specially designed primers for amplification of post-splicing HLA-B27 mRNA [25]. The post-splicing HLA-B27 mRNA was reverse transcripted to complementary ssDNA using the ABI sequence detection system (ABI 5700, Foster City, CA, USA) for measurement of the binding of SYBR green I fluorescence dye to the double-stranded DNA (dsDNA). The concept of threshold fluorescence is the basis of the real-time RT-PCR method. A threshold value (cycle threshold, CT) is determined by the number of cycles required to yield the fluorescence signal above the background fluorescence, which is usually 10 times the standard deviation of the baseline [30]. During PCR amplification, the number of molecules synthesized at CT (X_CT_) depends on the number of template molecules present at the beginning of the reaction (X_0_), the reaction efficiency (E), and the number of amplification cycles at CT [31]:

X_CT_ = X_0_·(1 + E)^CT^. (1)

As the number of molecules synthesized at CT is constant (C) for each type of reaction, then Equation (1) can be rearranged as following:

X_0_ = C·(1 + E)^−CT^. (2)

To correct for differences in DNA or RNA load between patient samples, a sample PCR should be normalized to a reference PCR [31]. In this study, a housekeeping gene “glyceraldehyde 3-phosphate dehydrogenase, GAPDH” was used. Housekeeping genes are present in all nucleated cell types, since they are necessary for basis cell survival and not linked to the biological response of the experimental treatment. Thus, the numbers of template molecules present at the beginning of the reaction for the sample and reference follow these relationships, respectively:

X_0,S_ = C·(1 + E)^−CT_S_^ (3)

X_0,R_ = C·(1 + E)^−CT_R_^. (4)

Combining Equations (3) and (4) and rearranging:

ΔCT = CT_S_ − CT_R_ = −[log(X_0,S_/X_0,R_)]/[log(1 + E)] (5)

Hence, relative quantitation of the HLA-B27 mRNA is related to ΔCT, the more the amount of HLA-B27 mRNA relative to GAPDH, the more negative the ΔCT value.

The primers for HLA-B27 mRNA were: sense strand 5′-CCTGG GCTGG CTCCC ACTC-3′ and antisense strand 5′-CACGT AGCCC ACGGT GATG-3′ with a size of 90 base pair (bp). The primers for GAPDH were: sense strand 5′ GAACA TCATC CCTGC ATCCA-3′ and antisense strand 5′-CCAGT GAGCT TCCCG TTCA-3′ with a size of 73 bp.

### 2.7. Analysis of H5 RNA Samples

The target ssRNA and the corresponding ssDNAs of avian H5N1 influenza virus were based on the conserved RNA sequence of hemagglutinin 5 (H5), with base sequences as following:

1. 60-mer target H5 ssDNA (60-mer, the underlined part is complementary to the probe),
  3′-GTTAA ATAAG TTGTC ACCGC TCAAG GGATC GTGAC CGTTA GTACC ATCGA CCAGA TAGAA-5′
2. 30-mer target H5 ssDNA (30-mer, perfect match to the probe),
  3′-AGGGA TCGTG ACCGT TAGTA CCATC GACCA-5′
3. 138-mer target H5 ssRNA (138-mer, the underlined part is complementary to the probe),
  5′-GUGUU UUUAA CUACA AUCUG AACUC ACAAA UUUAA AUGCA AAUUC UGCAU UGUAA CGAUC CAUUG GAGCA CAUCC AUAAA GAUAG ACCAG CUACC AUGAU UGCCA GUGCU AGGGA ACUCG CCACU GUUGA AUAAA UUG-3′

### 2.8. Analysis of HLA-B27 mRNA Samples

The HLA-B27 mRNA samples were prepared by extraction of total RNA from 3 mL of peripheral blood using a RNA Blood Mini Kits (QIAamp, Hilden, Germany). To perform FOPPR biosensing tests, the extracted RNA samples were diluted 20 times by the HB, then 200 μL of each diluted sample was injected into a sensor chip to obtain the sensor response. The base sequence of the HS-ssDNA probe for HLA-B27 mRNA detection is as follows:

Thiolated HLA-B27 ssDNA probe (19-mer base with 20-mer thymine spacer, MDBio, Taipei, Taiwan)
HS-(CH_2_)_6_-5′-T_20_-CCTGG GCTGG CTCCC ACTC-3′.

This study has been evaluated and approved by the ethics committee of Tzu Chi Hospital.

## 3. Results and Discussion

### 3.1. Construction of Sensor Layer

**Figure 2:**
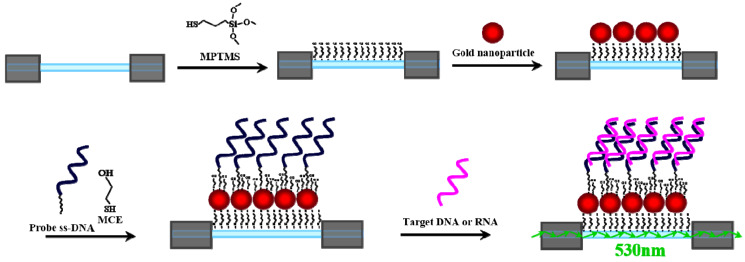
A schematic illustration of sensor layer construction: modification of MPTMS on fiber core surface, immobilization of AuNPs, bioconjugation of ssDNA probe, and hybridization of ssDNA probe with target ssDNA or ssRNA.

To detect the target ssDNA or ssRNA in a sample, a biosensing strategy based on conjugation of a ssDNA probe to the immobilized AuNPs on the fiber core surface was employed. Figure 2 shows a schematic of the strategy. A partially unclad optical fiber was functionalized with a thiol-terminated group by immersing in a toluene solution of 1% MPTMS. Then, the mercaptosilane-modified fiber was incubated in a solution of AuNPs to form a submonolayer of AuNPs on the fiber core surface. Next, the AuNP-modified fiber was first functionalized with a ssDNA probe, which was designed to bind specifically with its complementary target ssDNA or ssRNA. To minimize the non-specific binding and maximize hybridization efficiency between probe and target, a thiol-terminated alcohol, such as MCE and MCH [32,33,34], is often co-assembled with a thiolated ssDNA probe to form a mixed self-assembled monolayer (SAM) for erecting the ssDNA probe on AuNPs at a biocompatible interface with –OH groups to resist nonspecific adsorption. However, the treatment of thiol-terminated alcohols is tricky, too much thiolated alcohol will detach the HS-ssDNA probe from the AuNP surface, hence accurate control of reaction conditions is required [35].

To ensure maximum immobilization efficiency of the HS-ssDNA probes, it has been suggested when KH_2_PO_4_ concentration is greater than 0.4 M, maximum HS-ssDNA coverage was achieved due to minimized intermolecular electrostatic repulsion between neighboring stands of ssDNA under the high ionic strength conditions [33].

**Figure 3:**
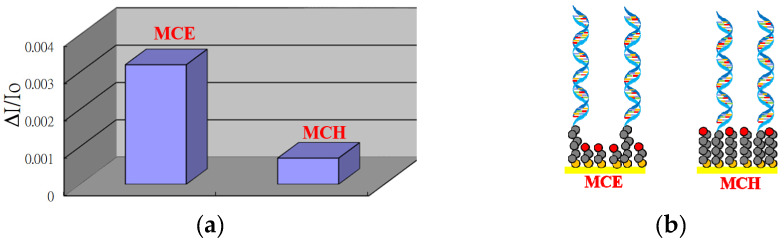
(**a**) Normalized absorbance changes before and after DNA hybridization for 27-mer ssDNA probe-conjugated AuNPs on glass slides based on 2-mercaptoethanol (MCE) or 6-mercaptohexanol (MCH) in the mixed SAM, (**b**) schematic illustration of mix SAMs with MCE and MCH as diluent.

Although, MCH has been frequently used as a diluent in a mixed SAM to minimize non-specific binding and maximize hybridization efficiency between probe and target [32,33,34], the length of MCH is comparable to that of the (CH_2_)_6_ spacer in the thiolated ssDNA probes. It has been suggested that a longer spacer between the ssDNA and the surface improves both, the limit of detection (LOD) and kinetics of DNA hybridization [36]. Hence, we explore and compare the hybridization behaviors of using MCE and MCH as diluents in mixed SAMs. Here a 27-mer thiolated ssDNA probe was used in both mixed SAMs using AuNP-modified glass slides as substrates. Upon DNA hybridization on the ssDNA-modified slides by adding the corresponding complementary target ssDNA, the increase in local RI on the AuNP surface results in the increase of absorbance of the AuNPs on both slides. As shown in Figure 3, the normalized absorbance change (ΔA/A_0_ = A_h_/A_0_ − 1, where A_0_ and A_h_ are the absorbances of the slide before, and after, DNA hybridization, respectively) of the slides with MCE in the mixed SAM is 4 times larger than that with MCH. This observation is consistent with a previous report [36], which attributes the difference of the two viewpoints. First, the greater the distance between the ssDNA probe and the surface, the less restricted the immobilized ssDNA probe, and hence, the hybridization efficiency. Second, a greater loss of ssDNA probe molecules with the longer mercaptoalkyl alcohol diluent MCH due to the greater van der Waals forces between longer diluent chains in the mixed SAM, and hence, higher driving force to remove the thiolated ssDNA probe molecules.

Following the previous report about the kinetics of MCH-mediated desorption of pre-adsorbed thiolated ssDNA probe molecules [37], we explore and optimize the reaction time for MCE-mediated desorption of pre-adsorbed thiolated ssDNA probe molecules. We had the MCE reaction times of 70 s, 5 min, and 1 h for comparison, the normalized absorbances (A_h_/A_0_) of the slide after DNA hybridization were 1.04, 1.06 and 1.06, respectively, as shown in Figure S2 and Table S1 of the Supplementary Material. According to the results, the MCE reaction time was chosen to be 5 min.

### 3.2. Label-Free Detection of DNA-DNA Hybridization

The hybridization affinities of thiolated ssDNA probes of various lengths (19, 21, 23, 25, 27-mer) toward the corresponding complementary target ssDNAs, and hence, their corresponding limits of detection (LODs, defined at S/N ratio = 3) using the FOPPR biosensor were evaluated. To ensure maximum hybridization efficiency, a high salt concentration hybridization buffer (HB) [33,38] was used to decrease the electrostatic repulsion between anionic chains of oligonucleotides and the target ssDNA.

**Figure 4:**
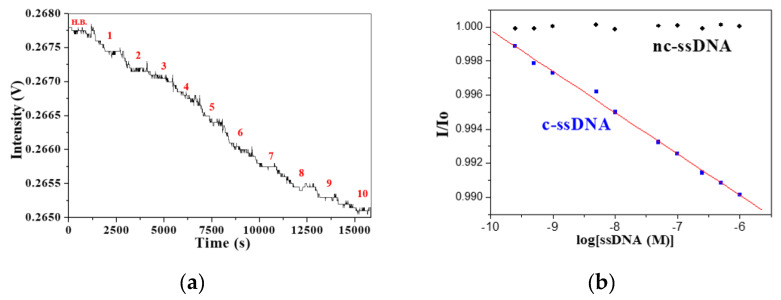
(**a**) A typical sensorgram using a 25-mer ssDNA probe for real-time detection of 25-mer target ssDNA upon sequential injection of standard solutions, where step 1 to step 10 represent concentrations 2.5 × 10^−10^, 5.0 × 10^−10^, 1 × 10^−9^, 5 × 10^−9^, 1 × 10^−8^, 5 × 10^−8^, 1.0 × 10^−7^, 2.5 × 10^−7^, 5.0 × 10^−7^, 1 × 10^−6^ M, respectively. (**b**) The plots of I/I_0_ versus target ssDNA concentration for both complementary ssDNA (c-ssDNA, blue square) and non-complementary ssDNA (nc-ssDNA, black diamond).

In order to test the detection levels of our FOPPR biosensor to analyze the DNA of various lengths, we measured the transmitted light intensity (I/I_0_) as a function of complementary ssDNA (c-ssDNA) concentration. Figure 4a shows a typical real-time FOPPR sensorgram with a 25-mer ssDNA probe to detect its corresponding 25-mer c-ssDNA over the concentration range from 2.5 × 10^−10^ to 1.0 × 10^−6^ M. With the increasing concentration of c-ssDNA, the amount of c-ssDNA hybridized at the sensor surface increases, resulting in increased local RI at the AuNP surface and increased absorbance of the AuNP submonolayer, and hence, the decrease of transmitted light intensity. As shown in Figure 4b, a plot of I/I_0_ versus c-ssDNA concentration is linear (*r* = −0.998) over a concentration range of about 4 orders. Taking the noise as the standard deviation of I/I_0_ for 5 repetitive measurements in buffer, a LOD of 1.1 × 10^−10^ M is calculated from the plot. A negative control experiment was carried out to test the specificity and anti-nonspecific adsorption ability of the mixed SAM with respect to non-specific ssDNA binding. As shown in Figure 4b, the I/I_0_ value remains unchanged with increasing concentration of non-complementary ssDNA (nc-ssDNA) concentration over the concentration range from 2.5 × 10^−10^ to 1.0 × 10^−6^ M, suggesting our FOPPR biosensor is capable of performing sequence specific analysis.

Although DNA hybridization is fairly well-understood in bulk solution, the molecular interactions, and hence, the binding affinity is more complex at the surface. The availability of real-time FOPPR kinetic data may provide insight about binding affinity and binding kinetics of two complementary ssDNAs of various lengths at the surface [8,39]. Using an approach as previously described [8], Table 2 lists the calculated binding constants for perfectly matched hybridization of oligonucleotides with lengths of 19, 21, 23, 25, 27-mer. It can be seen that the binding constant (K_a_) increases with the length of the oligonucleotide pairs. A plot of log(K_a_) versus oligonucleotide length (*L*) is reasonably linear (*r* = 0.963) which follows the following equation: log(K_a_) = 0.122 × *L* + 4.65. These binding constants of surface hybridization reactions are consistent with those reported by other research groups [36,40,41,42], which are orders of magnitude smaller than those of solution hybridization under the same conditions [41]. It has been suggested the much lower surface binding constants might reflect electrostatic and steric penalties associated with penetration of a target strand into a probe layer [43]. Due to the higher affinity with longer oligonucleotide length, the LOD also improves as oligonucleotide length increases, as shown in Table 1. This may be attributed to the following two reasons. First, higher K_a_ leads to a higher amount of c-ssDNA hybridized at the surface at the same cDNA concentration. Second, with the same amount of c-ssDNA hybridized at the surface, the longer oligonucleotide pairs result in a greater change of local RI at the AuNP surface. Both effects lead to increased absorbance of the AuNP submonolayer, and hence the decrease of transmitted light intensity.

### 3.3. Label-Free Detection of DNA-RNA Hybridization Using H5 Conserved Sequence as Model

**Figure 5:**
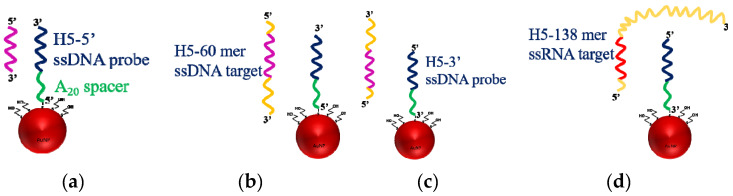
Schematic representation of the interaction between, (**a**) 30-mer H5-5′ ssDNA probe immobilized at 5′-end and 30-mer target ssDNA, (**b**) 30-mer H5-5′ ssDNA probe immobilized at 5′-end and 60-mer target ssDNA, (**c**) 30-mer H5-3′ ssDNA probe immobilized at 3′-end and 60-mer target ssDNA, and (**d**) 30-mer H5-3′ ssDNA probe immobilized at 3′-end and 138-mer target RNA.

**Figure 6:**
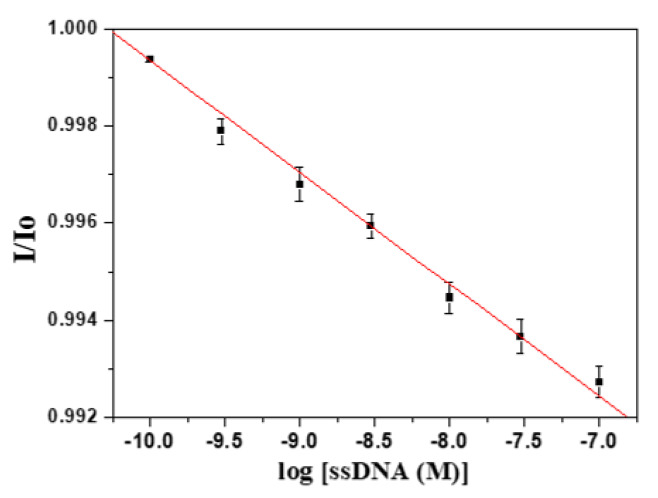
Calibration graph of a FOPPR biosensor using a 30-mer H5-5′ ssDNA probe immobilized at 5′-end in response to 30-mer target H5 ssDNA (n = 5).

Avian H5N1 influenza virus has received enormous attention as a potential pandemic threat and also a threat to human health. H5N1 virus is a negative single strand RNA (ssRNA) virus, and has a highly conserved sequence in the hemagglutinin genes. We chose the conserved RNA sequence of hemagglutinin 5 (H5) as a model ssRNA to test our FOPPR biosensor for label-free detection of DNA-RNA hybridization. The H5 ssDNA probe used is a 30-mer thiolated ssDNA with a 20-mer adenine spacer. To investigate the steric effect, we first designed ssDNA as the target, with only the 30-mer hybridization region, as shown in Figure 5a. As shown in Figure 6, a calibration plot of I/I_0_ versus ssDNA concentration is linear (*r* = −0.997) with a LOD of 1.9 × 10^−10^ M calculated from the plot. The K_a_ is calculated to be 9.8 × 10^8^ M^−1^, which is similar to that of the DNA-DNA counterpart and such results are consistent with a previous report [44].

**Figure 7:**
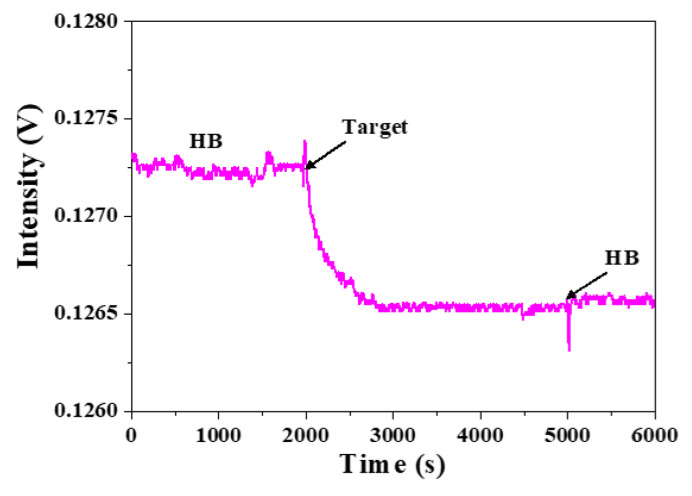
A typical real-time FOPPR sensorgram with H5-5′ ssDNA probe to detect 60-mer target ssDNA at a concentration of 1.0 × 10^−6^ M.

To consider the steric effect when a target ssDNA has a length longer than the ssDNA probe, we divide this into two possibilities as shown in Figure 5b,c, where the hybridization region is located unevenly in the target ssDNA. In this scenario, we separately thiolate the H5 ssDNA probe at either 3′-end to produce thiolated H5-3′ ssDNA probe or 5′-end to produce thiolated H5-5′ ssDNA probe so that the orientation of the immobilized ssDNA probes are opposite. As a result, the same 60-mer target ssDNA will bind with the two immobilized ssDNA probes in opposite directions, as shown in Figure 5b,c. When H5-5′ ssDNA probe, as shown in Figure 5b, is used, the longer fragment (not used for hybridization) becomes close to the surface and may cause more steric hindrance than when H5-3′ ssDNA probe as shown in Figure 5c is used. Figure 7 shows a typical real-time FOPPR sensorgram, when H5-5′ ssDNA probe was used to detect the 60-mer target ssDNA, at a concentration of 1.0 × 10^−6^ M. A response time of ~650 s was observed. We carried out the binding affinity study as described in Section 3.2 to examine the binding constants and LODs in these two cases. Results as listed in Table 3 show that the K_a_’s are 1.3 × 10^7^ M^−1^ and 2.9 × 10^8^ M^−1^ when H5-5′ ssDNA probe, and H5-3′ ssDNA probe are used, respectively. In addition, the LODs are 1.4 × 10^−8^ M and 1.2 × 10^−10^ M when H5-5′ ssDNA probe, and H5-3′ ssDNA probe are used, respectively.

As shown in Table 3, the binding affinity data suggest that steric hindrance is the least when the target ssDNA has the same length as the ssDNA probe, while the steric hindrance is more serious when the longer fragment not for hybridization is close to the surface. Nevertheless, as shown in Table 3, the LOD in the case, as shown in Figure 5c, is similar to, or slightly better, than that in the case as shown Figure 5a. This may be attributed to the larger size of the analyte, which causes larger change in local RI upon hybridization.

**Figure 8:**
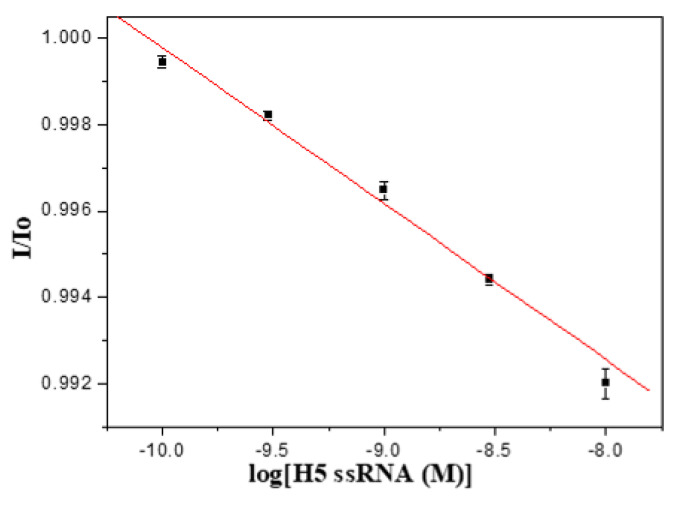
A calibration plot of H5-3′ ssDNA probe to detect 138-mer target ssRNA, ranging from 1.0 × 10^−^^10^ M to 1.0 × 10^−^^8^ M, (n = 3).

The H5N1 influenza virus is a (−)RNA virus, therefore we employed one anti-sense synthetic H5 ssRNA with 138 nucleotides for further exploration of the feasibility of label-free detection of DNA-RNA hybridization. In this study, we employed the thiolated H5-3′ ssDNA probe for detection of the 138-mer target H5 ssRNA. As shown in Figure 8, a calibration plot of I/I_0_ versus ssRNA concentration is linear (*r* = −0.994) with a LOD of 1.1 × 10^−10^ M calculated from the plot. The K_a_ is calculated to be 6.4 × 10^8^ M^−1^, which is similar to that of the DNA-DNA counterpart, and such results are consistent with a previous report [44].

### 3.4. Label-Free Detection of HLA-B27 mRNA in Real Clinical Samples and Comparative Study by Real-Time RT-PCR

From statistic of epidemiology, 90% AS patients exhibit higher expression of HLA-B27 mRNA [45]. In this study, the choice of a 19-mer ssDNA probe for HLA-B27 mRNA detection is based on published data of HLA-B27 gene sequence, where there are 4 exons in the gene structure: 58–130 of exon 1, 259–528 of exon 2, 773–1048 of exon 3, and 1624–1899 of exon 4. We chose the mRNA sequence across the tail of exon 1 and the beginning of exon 2, but not that of the original DNA nucleotide sequence, to design the HLA-B27 ssDNA probe, which is a sense strand with base sequence: 5′-CCTGG GCTG (sequence 122–130 in exon 1) GCTCC CACTC (sequence 259-268 in exon 2)-3′ having thiol group bonded to the immobilized AuNPs on the fiber core surface and the ssDNA immobilized at the 5′ end. To minimize steric effect, oligo-dT 20, a string of 20 deoxythymidylic acid, was employed as a spacer between the ssDNA and the thiol group in the HLA-B27 ssDNA probe.

**Figure 9:**
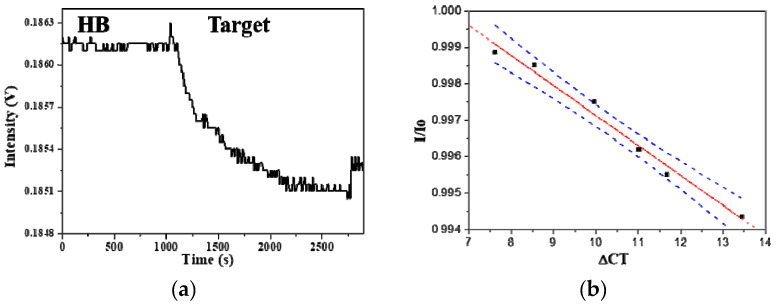
(**a**) Real-time FOPPR sensorgram with HLA-B27 ssDNA probe to detect mRNA in a sample which exhibits a ΔCT value of 13.64; (**b**) Graph displaying the correlation of results between the FOPPR biosensor and the real-time RT-PCR method for detection of HLA-B27 mRNA in blood samples. Blue dashed lines are the 95% confidence intervals for prediction with the linear regression model.

Six blood samples were collected from patients with ankylosing spondylitis (AS) by Buddhist Dalin Tzu Chi General Hospital. Total RNA was extracted from each sample and diluted 20 times with the HB to minimize the RI difference between the sample and the blank. Figure 9a shows a representative sensorgam when a diluted HLA-B27 mRNA sample was injected into a sensor chip, a response time of about 1100 s and a steady state signal can be observed within 20 min. Such an analysis time is much shorter than that required by real-time RT-PCR (about 2 h).

The accuracy of the results by the FOPPR biosensor for the analysis of HLA-B27 mRNA was compared with that by the real-time RT-PCR method using six extracted blood samples from AS patients. As shown in Figure 9b, comparison of the results by the two methods performed by linear correlation analysis shows a correlation coefficient of 0.991 and a p value < 0.0001 at the 95% level of significance, indicating that the results arising from both methods align. Since a relationship between B27 gene expression and patients’ clinical disease activity was established [25], quantitation of HLA-B27 mRNA may be useful as an indicator for monitoring the disease. Thus, the FOPPR biosensor provides a rapid and simple alternative to the laborious and time-consuming real-time RT-PCR method.

## 4. Conclusions

This work demonstrates the feasibility of the FOPPR biosensor, employing ssDNA probe for sensitive and label-free detection of nucleic acids, including DNA and RNA oligonucleotides. The detection of the hybridization adsorption of short, unlabeled RNA and DNA oligonucleotides onto the AuNPs on the fiber core surface, at concentrations at the sub-nM level, has been observed. This makes detection of target oligonucleotides as low as 1 fmol possible in the 10-μL sensor chip when the detection is taking place at a concentration near the LOD at the 10^−^^10^ M level. The sensor fibers could easily be regenerated and were used for multiple testing. Through exploration of the designs of the ssDNA probes we can conclude: (1) A longer spacer between the ssDNA in the probe and the surface improves both the LOD and hybridization affinity between DNA-DNA and DNA-RNA; (2) hybridization affinity increases with the length of the oligonucleotide pairs; (3) hybridization affinity between DNA-RNA is similar to that between DNA-DNA; (4) for target oligonucleotides where the hybridization region is located unevenly, steric hindrance is more serious, when the longer fragment, not for hybridization, is close to the surface.

The applicability of the FOPPR biosensor in real samples is also demonstrated by its application to detection of HLA-B27 mRNA in patients with AS. The results from six extracted blood samples indicate that the FOPPR sensor responses correlate well with the ΔCT values obtained by the real-time RT-PCR method. In summary, by combining the desirable features of label-free, real-time, and simple optics of the FOPPR biosensor with good surface chemistry, development of a sensitive, rapid, and a cost-effective nucleic acid biosensor has been demonstrated. Our FOPPR biosensor has been successfully applied to detection of protein biomarkers in real samples. However, for certain diseases, the concentration of target molecules/pathogens are at extremely low level. With the adoption of our recently developed fiber optic nanogold-linked immunosorbent assay (FONLISA) in FOPPR biosensor which exhibits fM sensitivity [15], we believe that the FOPPR biosensing equatmethodology can also be applied to the detection of SARS-CoV-2, a positive-sense single-stranded RNA (ssRNA) virus, which has resulted in the recent global COVID-19 outbreak, and will continue to broaden the application fields in molecular biology and biomedical diagnosis.

## Figures and Tables

**Table 1:** Sequences of ssDNA probes and target ssDNAs used in this study.

Name	Sequence (5′→3′), functional at 5′ end: HS-(CH_2_)6-ssDNA
19-mer probe	CCTGG GCTGG CTCCC ACTC
21-mer probe	AGATC AGTGC GTCTG TACTA G
23-mer probe	AGATC AGTGC GTCTG TACTA GCA
25-mer probe	AGATC AGTGC GTCTG TACTA GCACA
27-mer probe	ATGGG CGGCA TGAAC CGGAG GCCCA TC
Name	Sequence (3′→5′)
19-mer target	GGACC CGACC GAGGG TGAG
21-mer target	TCTAG TCACG CAGAC ATGAT C
23-mer target	TCTAG TCACG CAGAC ATGAT CGT
25-mer target	TCTAG TCACG CAGAC ATGAT CGTGT
27-mer target	TACCC GCCGT ACTTG GCCTC CGGGT AG
27-mer target (nc) ^1^	CTAGC TAGCT AGCTA GCTAG CTAGC TA

^1^ nc: Non-complementary.

**Table 2:** LOD and K_a_ comparison obtained by FOPPR biosensor with ssDNA probes of different oligonucleotide lengths.

Oligonucleotide Length (bp)	Molecular Weight (Da)	Binding Constant (K_a_)	LOD
19-mer	5912.9	7.2 × 10^6^ M^−^^1^	2.4 × 10^−^^9^ M
21-mer	6657.5	2.5 × 10^7^ M^−^^1^	4.9 × 10^−^^10^ M
23-mer	7259.8	2.7 × 10^7^ M^−^^1^	3.3 × 10^−^^10^ M
25-mer	7862.2	4.5 × 10^7^ M^−^^1^	1.1 × 10^−^^10^ M
27-mer	8531.6	8.8 × 10^7^ M^−^^1^	9.6 × 10^−^^11^ M

**Table 3:** LOD and K_a_ comparison obtained by FOPPR biosensor with different combinations of ssDNA probe and target as shown in Figure 5.

Probe	H5-5’ ssDNA Probe		H5-3’ ssDNA Probe	
**Target**	30-mer ssDNA	60-mer ssDNA	60-mer ssDNA	138-mer RNA
**LOD**	1.9 × 10^−^^10^ M	1.4 × 10^−^^8^ M	1.2 × 10^−^^10^ M	1.1 × 10^−^^10^ M
**K_a_**	9.8 × 10^8^ M^−^^1^	1.3 × 10^7^ M^−^^1^	2.9 × 10^8^ M^−^^1^	6.4 × 10^8^ M^−^^1^

## Supplementary Materials

The following are available online at https://www.mdpi.com/1424-8220/20/11/3137/s1, Figure S1: AuNP images obtained from electron microscopy, Figure S2: UV-visible spectra of thiolated ssDNA/MCE-modified AuNPs on glass slides before and after DNA hybridization with various MCE reaction time, Table S1: Normalized absorbances of thiolated ssDNA/MCE-modified AuNPs on glass slides before and after DNA hybridization with various MCE reaction time.
