# Supplementary material for: Fiber Optic Particle Plasmon Resonance Biosensor for Label-Free Detection of Nucleic Acids and Its Application to HLA-B27 mRNA Detection in Patients with Ankylosing Spondylitis
Source: Sensors (Basel). 2020 Jun 1;20(11):3137. doi: 10.3390/s20113137 (PMC7309088; doi:10.3390/s20113137)
Supplement: Supplementary file 1 [file sensors-20-03137-s001.pdf]

Supplementary Material

Fiber Optic Particle Plasmon Resonance Biosensor for Label-Free Detection of Nucleic Acids and Its Application to HLA-B27 mRNA Detection in Patients with Ankylosing Spondylitis

Yen-Ta Tseng [1,†], Wan-Yun Li [1,†], Ya-Wen Yu [1,†], Chang-Yue Chiang [1,4], Su-Qin Liu [2], Lai-Kwan Chau [1,*], Ning-Sheng Lai [2,*], Cheng-Chung Chou [3,*]

1. Department of Chemistry and Biochemistry and Center for Nano Bio-Detection, National Chung Cheng University, Chiayi 62102, Taiwan; tsengyentaozzy@gmail.com (Y.T.T.); smilelili72@gmail.com (W.Y.L.); wen19860312@hotmail.com (Y.W.Y.)
2. Immunology and Rheumatology, Department of Medicine, Buddhist Dalin Tzu Chi General Hospital, Chiayi 62247 Taiwan; df897226@tzuchi.com.tw (S.Q.L.)
3. Department of Biomedical Sciences, National Chung Cheng University, Chiayi 62102, Taiwan
4. Graduate School of Engineering Science and Technology, National Yunlin University of Science and Technology, Yunlin 64002, Taiwan; chiangcy@yuntech.edu.tw (C.-Y.C.)

* Correspondences: chelkc@ccu.edu.tw (L.K.C.); Q12015@tzuchi.com.tw (N.S.L.); bioccc@ccu.edu.tw (C.C.C.)
Tel.: +886-5-2729377 (L.K.C.); +886-5-264-8000 ext. 5003 (N.S.L.); +886-5-2720411 ext. 66506 (C.C.C.)

† These authors contributed equally to this work.

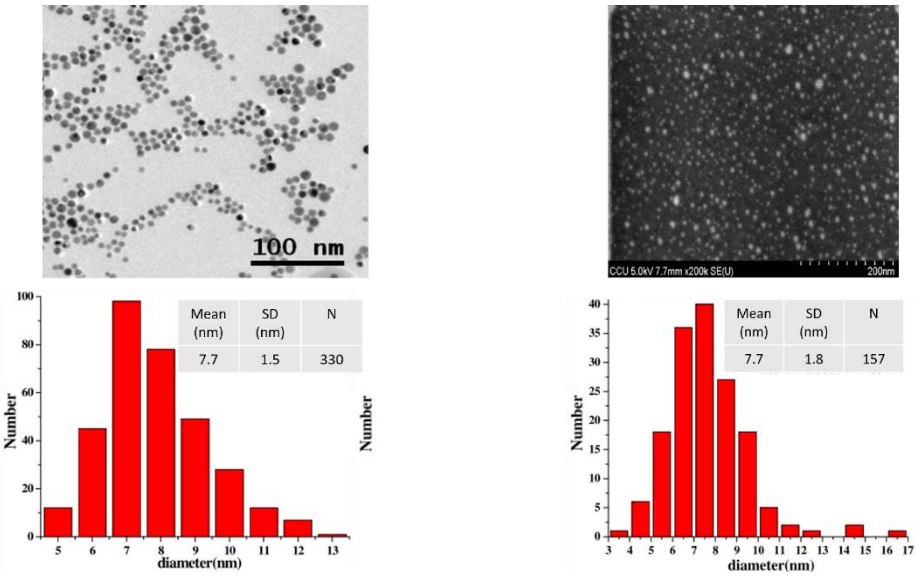

(a) (b)

Figure S1. AuNP images obtained from electron microscopy. (a) A TEM image of synthesized AuNPs, the mean size of AuNPs is 7.7 ± 1.5 nm. (b) A SEM image of AuNPs on an optical fiber, the mean size of AuNP is 7.7 ± 1.8 nm.

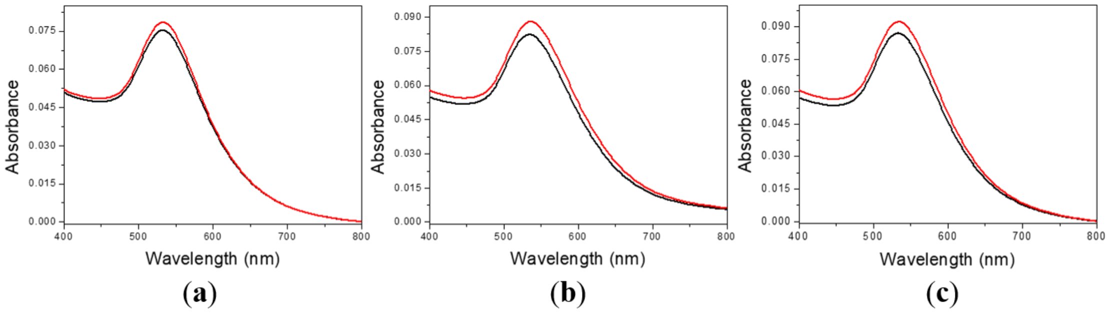

Figure S2. UV-visible spectra of thiolated ssDNA/MCE-modified AuNPs on glass slides before (black line) and after (red line) DNA hybridization with MCE reaction time at (a) 70 s; (b) 5 min; and (c) 1 h.

Table S1. Normalized absorbances of thiolated ssDNA/MCE-modified AuNPs on glass slides before and after DNA hybridization at 532 nm with various MCE reaction times.

MCE time	Before hybridization	After hybridization	A_h/A_0
70 s	0.075	0.078	1.04
**5 min**	**0.076**	**0.081**	**1.06**
1 h	0.087	0.092	1.06
